# Supplementary material for: Causal Relationship between Aging and Anorexia Nervosa: A White-Matter-Microstructure-Mediated Mendelian Randomization Analysis
Source: Biomedicines. 2024 Aug 16;12(8):1874. doi: 10.3390/biomedicines12081874 (PMC11351342; doi:10.3390/biomedicines12081874)
Supplement: Supplementary file 1 [file biomedicines-12-01874-s001.zip › Supplementary Figure/Figure S7/Single SNP plot of MR analysis for ubm-b-1914 to AN.pdf]

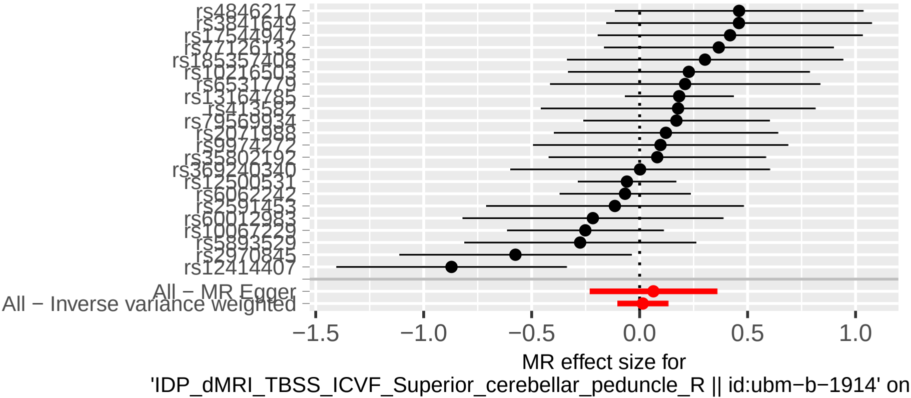

rs4846217
rs3841649
rs17544947
rs77126132
rs185357408
rs10216503
rs6531779
rs13164785
rs413582
rs79569934
rs2071988
rs9974272
rs35802192
rs369240340
rs12500531
rs6062242
rs2591453
rs60012983
rs10067229
rs5893529
rs2970845
rs12414407
All − MR Egger
All − Inverse variance weighted
−1.5
−1.0
−0.5
0.0
0.5
1.0
MR effect size for
'IDP_dMRI_TBSS_ICVF_Superior_cerebellar_peduncle_R || id:ubm−b−1914' on
